# Circulating microRNA-122 as Potential Biomarker for Detection of Testosterone Abuse

**DOI:** 10.1371/journal.pone.0155248

**Published:** 2016-05-12

**Authors:** Olivier Salamin, Laetitia Jaggi, Norbert Baume, Neil Robinson, Martial Saugy, Nicolas Leuenberger

**Affiliations:** Swiss Laboratory for Doping Analyses, University Center of Legal Medicine, Lausanne and Geneva, Centre Hospitalier Universitaire Vaudois and University of Lausanne, Lausanne, Switzerland; Gustave Roussy, FRANCE

## Abstract

MicroRNAs (miRNAs) are small non-coding RNAs that regulate gene expression and thus influence many cellular and physiological processes. miRNAs are also present in cell-free body fluids such as plasma or serum, and these circulating miRNAs are very stable, sensitive, and specific biomarkers of pathophysiological states. In this study, we investigated whether circulating miRNAs could serve as biomarkers of exogenous testosterone administration. Misuse of testosterone as a performance-enhancing drug is thought to be widespread in sports. Detection of testosterone through the urinary steroid profile of the Athlete Biological Passport faces several obstacles, indicating that new biomarkers are required. To this end, we analyzed plasma miRNA levels by high-throughput quantitative real-time PCR. Plasma samples were obtained before and at several time points after transdermal and oral testosterone administration. Screening identified three potential candidate miRNAs that were altered by both routes of testosterone administration. Longitudinal monitoring of these candidates revealed that variation in two of them (miR-150 and miR-342), relative to the corresponding levels in control samples, was testosterone-independent. However, levels of the liver-specific miR-122 increased 3.5-fold 1 day after drug intake. Given that testosterone is metabolized by the liver, this observation suggests that miR-122 in cell-free fluids may be used as a sensitive biomarker of testosterone misuse via multiple dosing routes and could therefore be integrated into a blood-based multiparametric follow-up.

## Introduction

Testosterone, the principal endogenous androgenic anabolic steroid (EAAS), is secreted by endocrine glands. It regulates many physiological processes in adult males, including muscle protein metabolism, sexual and cognitive functions, erythropoiesis, hepatic lipid metabolism, and bone metabolism [1, 2]. Although its use in sports is prohibited by the World Anti-Doping Agency (WADA), testosterone is widely abused by athletes as a performance-enhancing drug [3]. In addition to their anabolic action, small doses of anabolic-androgenic steroids can also lower fatigue levels, and accelerate recovery, leading to a higher training load and faster increase in physical performance [4].

In doping control, screening for testosterone (T) misuse is primarily performed by GC-MS and GC-MS/MS through assessment of a urinary steroid profile that includes T, its precursors, and its metabolites. For several decades, evaluation of the ratio of testosterone to epitestosterone (T/E) has been the gold standard for screening for T administration. Based on population references, a cut-off value of 4 was established as the threshold for this ratio [5]. When the ratio exceeds the threshold, urine samples are submitted for confirmation analysis with GC-C-IRMS [4]. However, despite its sensitivity and specificity, the T/E biomarker suffers from a short detection window, particularly when T is taken orally, as well as high inter-individual variability due to natural high T/E and genetic polymorphism [6–8]. To overcome these limitations and improve the ability to detect T, the urinary steroidal module of the Athlete Biological Passport (ABP) was recently implemented (January 1, 2014). This module relies on a longitudinal/multiparametric approach using specific markers of altered metabolism of endogenous steroidal hormones, including T/E and other metabolites ratios (Androsterone (A)/T, A/Etiocholanolone (Etio), 5α-Adiol/5β-Adiol), for each athlete using intra-individual references generated by a Bayesian Adaptive Model [9].

However, more than 1 year after its implementation, the efficacy of the steroidal module of the ABP is still in question, principally due to instability of urine samples (bacterial contamination, medications, enzyme induction-inhibition, etc.) and polymorphism of the *UGT2B17* gene, which encodes a protein responsible for testosterone glucuronidation [10–12]. Therefore, new approaches and potential biomarkers of T abuse must be investigated to improve the discriminative performance of the ABP.

MicroRNAs (miRNAs), a class of small non-coding RNAs about 22 nucleotides in length [13], regulate gene expression post-transcriptionally by decreasing mRNA stability or inhibiting translation of mRNA into protein. Consequently, miRNAs are implicated in many cellular processes such as differentiation, proliferation, metabolism, and apoptosis [14, 15]. Recently, miRNAs were detected in various body fluids, including urine, plasma, serum, saliva, and tears [16]. Although the physiological functions and regulation of these so-called ‘circulating miRNAs’ are largely unknown, they are associated with specific pathophysiological states such as cancers and cardiovascular diseases [17–19]. In an anti-doping context, multiple groups reported associations between altered plasma miRNA expression profiles and doping interventions such as erythropoiesis-stimulating agents (ESA), autologous blood transfusion, and recombinant growth hormone (rhGH) administration [20–23]. In comparison with proteins, circulating miRNAs offer non negligible advantages as non-invasive biomarkers. miRNAs are highly stable in body fluids, and are resistant to RNases, fluctuations in pH, and to multiple-freeze/thaw cycles [24, 25]. In addition, expression of many miRNAs is specific to a tissue or cell type [26], and their levels can be easily measured using common laboratory techniques, including amplification-associated methods that require very small sample volumes [27].

Therefore, in this study we investigated whether circulating miRNAs can be used as surrogate biomarkers of testosterone abuse via various dosing routes. We compared the resultant data with both commonly measured parameters and emerging parameters.

## Material and Methods

### Clinical study

All samples were derived from a previous clinical study that included 19 healthy male volunteers aged 19–28 (mean 24.3 ± 2.7 years) with BMI between 18.3 and 27.2 kg/m^2^ (mean 23.1 ± 2.4) and different *UGT2B17* genotypes (ins/ins, ins/del, and del/del) [6]. All subjects gave signed consent form and the protocol was authorized by the Ethical Commission for the Clinical Research of the Faculty of Biology and Medicine (University of Lausanne, Switzerland) and Swissmedic (Protocol no. 155/11). The clinical trial took place over 5 weeks, divided into four major phases (S1 Fig): the first week was dedicated to collection of control samples (no treatment), followed by the administration of two transdermal systems that delivered 2.4 mg/24 h (Testopatch^®^, Pierre Fabre Pharma GMBH, Freiburg, Germany) during the second week. After a wash-out period of 2 weeks, two 40 mg testosterone undecanoate (TU) tablets (Andriol Testocaps^®^, Essex Chemie AG, Luzern, Switzerland) were ingested by each volunteer. First blood collection was performed at 07:00 AM. Plasma and serum samples were centrifuged at 1500 x g for 15 min after 15 min stabilization at room temperature. Plasma and serum aliquots were kept at -20°C until analyses as described in [6].

### miRNA quantitative real-time PCR (qRT-PCR)

Total RNA, including miRNAs, was isolated from 200 μl of plasma using the miRCURY RNA Isolation Kit—Biofluids (Exiqon, Vedbaek, Denmark) with minor modifications. Briefly, before RNA extraction, all plasma or serum samples were thawed completely, followed by centrifugation at 16,000 × g for 10 min to remove remaining cell debris and platelets [28, 29]. During the treatment with lysis buffer, 1 μl of spike-in mix containing synthetic UniSp2, UniSp4, and Unisp5 and 1 μl of cel-miR-39-3p (RNA Spike-in Kit, Exiqon) were added to the samples as a control for extraction efficiency. After protein precipitation, supernatant was loaded onto spin columns. Finally, the purified RNA was eluted with 35 μL RNAse free-water.

For miRNA profiling, 4 μL of eluted RNA was used in a 20-μL reverse transcription (RT) reaction. UniSp6 Spike-in miRNA was added to the RT reaction mix. The resulting cDNA was diluted 110 times and profiled for the relative abundance of 372 miRNAs using miRNA Ready-to-Use PCR, Human panel I, V4.R quantitative real-time PCR (qRT-PCR) arrays (Exiqon), as described in [21]. Raw data were analyzed using the LightCycler 480 software (version 1.5.0). All primer sequences are available on the Exiqon web site (http://www.exiqon.com/mirna-pcr-primer).

### Hematology, clinical chemistry, and immunology

Blood parameters were analyzed in EDTA samples using a fully automated hematology analyzer (Sysmex XT-2100i, Sysmex, Norderstedt, Germany). Free T was measured in serum using Immulite technology (Siemens AG, Munich, Germany). Alanine aminotransferase (ALT), aspartate aminotransferase (AST), and C-reactive protein (CRP) serum levels were measured using a Dimension EXL200 automated system (Siemens Healthcare Diagnostic SA, Zurich, Switzerland).

### Statistics

Unless specified otherwise, data are expressed as means ± SEM versus baseline. Statistical comparisons were performed using the two-tailed Student’s t-test (for free T) or non-parametric Wilcoxon signed-rank test (for miRNA levels). Differentially expressed miRNAs were identified using the R package LIMMA as described in [21]. P < 0.05 was considered to represent a statistically significant difference. Average Ct corresponds to the mean of Ct values at 0 and 24 h. Fold-Change (FC) was calculated from Ct values. Conditions were compared by one-way ANOVA (the *aov* function in R) and post-hoc pairwise comparisons were performed with Tukey’s Honestly Significant Difference (the *TukeyHSD* function in R). Descriptive and statistical comparisons were performed using various softwares (Microsoft Office Excel 2007, StataIC, StataCorp and R).

## Results

To investigate the utility of circulating miRNAs as biomarkers of testosterone misuse, miRNA profiling was performed by RT-PCR in samples obtained before (0 h) and at various time points after (12 and 24 h) oral and transdermal T administration (S1 Table). Data were analyzed using a linear model that paired values by subjects. Out of 372 miRNAs tested, 134 (including controls) were detected after ingestion of two TU tablets, and 143 were detected after application of diffusion patches. This screen enabled to identify the circulating miRNAs that were most affected by T administered by different routes (Table 1 and S2 Table). These candidates were selected for further investigations.

**Table 1:** miRNAs most affected by testosterone. List of the 12 most affected miRNAs 1 day after ingestion of two TU tablets or application of two Testopatches, identified by miRNA expression profiling.

	Oral Testosterone						Transdermal Testosterone				
miRNA	Fold-Change (FC)	FC.low	FC.high	Average Ct	P.value	miRNA	Fold-Change (FC)	FC.low	FC.high	Average Ct	P.value
**hsa-miR-122-5p**	**2.2**	**-1.07**	**5.17**	**30.24**	**0.015168**	**hsa-miR-122-5p**	**2.3**	**1.34**	**3.95**	**30.45**	**0.000812**
hsa-miR-146b-5p	-2.1	-3.66	-1.21	35.08	0.002894	hsa-miR-425-5p	-1.65	-2.63	-1.04	30.52	0.006124
hsa-miR-629-5p	-2.39	-4.04	-1.41	34.15	0.002396	hsa-miR-324-3p	-1.76	-2.48	-1.25	32.74	0.002622
hsa-miR-29b-3p	-2.51	-4.5	-1.4	33.91	0.001766	hsa-miR-582-5p	-1.97	-3.76	-1.04	34.76	0.005832
hsa-miR-29c-3p	-2.64	-5.76	-1.21	32.1	0.003952	hsa-let-7c-5p	-2	-3.92	-1.02	34.68	0.007064
hsa-miR-874-3p	-2.93	-6.3	-1.36	33.99	0.002421	hsa-let-7g-5p	-2.1	-4.56	1.03	28.93	0.009684
hsa-miR-361-5p	-2.99	-5.58	-1.6	31.47	0.002397	hsa-miR-423-5p	-2.13	-3.57	-1.27	31.59	0.001973
hsa-miR-140-3p	-3.16	-9.05	-1.11	28.91	0.006288	hsa-miR-29a-3p	-2.95	-9.75	1.12	30.99	0.009417
hsa-miR-423-3p	-3.21	-8.84	-1.17	31.83	0.010688	**hsa-miR-342-3p**	**-2.99**	**-9.08**	**1.02**	**28.86**	**0.005837**
hsa-miR-29a-3p	-3.59	-8.82	-1.46	31.36	0.000905	hsa-miR-338-3p	-3.07	-7.59	-1.24	33.78	0.001644
**hsa-miR-342-3p**	**-5.7**	**-13.93**	**-2.33**	**28.15**	**0.000228**	hsa-miR-424-5p	-3.36	-9.89	-1.14	32.7	0.002452
**hsa-miR-150-5p**	**-6.87**	**-21.08**	**-2.24**	**27.23**	**0.000295**	**hsa-miR-150-5p**	**-3.96**	**-15.47**	**-1.02**	**26.85**	**0.005019**
hsa-miR-486-5p	-1.29	-2.3	1.39	27.35	0.3626	hsa-miR-486-5p	-1.14	-1.7	1.31	27.78	0.5245

*miRNAs selected for individual assays are indicated in bold and miR-486-5p was chosen as an endogenous control for data normalization.

Significant variations in the levels of candidate miRNAs were only observed 24 h after testosterone intake via either route. Most circulating miRNAs decreased >2-fold at 24 h versus 0 h after administration; of these, miR-342 and miR-150 were the only miRNAs that were significantly affected by oral and transdermal T administration. By contrast, miR-122 was the only miRNA whose abundance increased under both conditions (2.3-fold on average). These miRNAs were selected for further analyses because of their abundance, statistical significance and fold change.

Profiling of circulating miRNAs as biomarkers of T also permitted to identify miRNAs that were not responsive to the treatment (S3 Table). Among these, miR-486-5p exhibited a low coefficient of variation across all samples under both conditions and was present at high levels in plasma. Therefore, miR-486-5p was selected as an internal control for RT-PCR validation.

**Fig 1:**
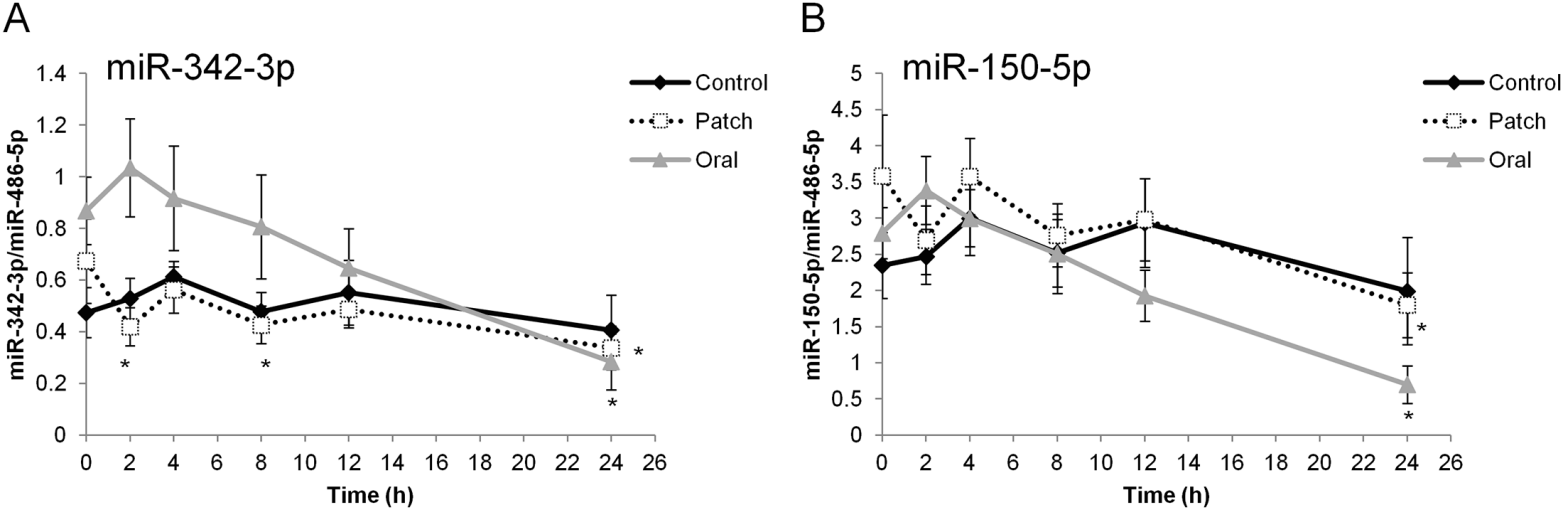
Circulating miRNA levels after oral and transdermal administration of T and during the control period. Levels of miR-342 (A) and miR-150 (B) in plasma samples collected from healthy volunteers after administration of oral (gray line) or transdermal (dashed line) T or in the control phase (black line). Collection time points are indicated on the x-axis. Data were normalized against the corresponding levels of endogenous miR-486-5p. Values are expressed as means (±SE) of 19 independent samples. (*) indicates statistically significant difference relative to time = 0 h.

The results of the screen were confirmed by individual assays throughout the timeline, with special emphasis on the three miRNAs identified as candidate markers of T administration. Measurements of miR-342 and miR-150 at the various time points confirmed the screening results: the levels of both miRNAs decreased significantly 24 h after T administration relative to their levels at 0 h (prior to T administration) (Fig 1). For miR-342, the decrease was also significant 2 and 8 h after transdermal T application (Fig 1A). However, when observing the kinetics of these two miRNAs during the control period, we noticed that the curves resembled those of the T periods. The three conditions differed primarily in regard to the baseline levels of miR-342 and miR-150.

**Fig 2:**
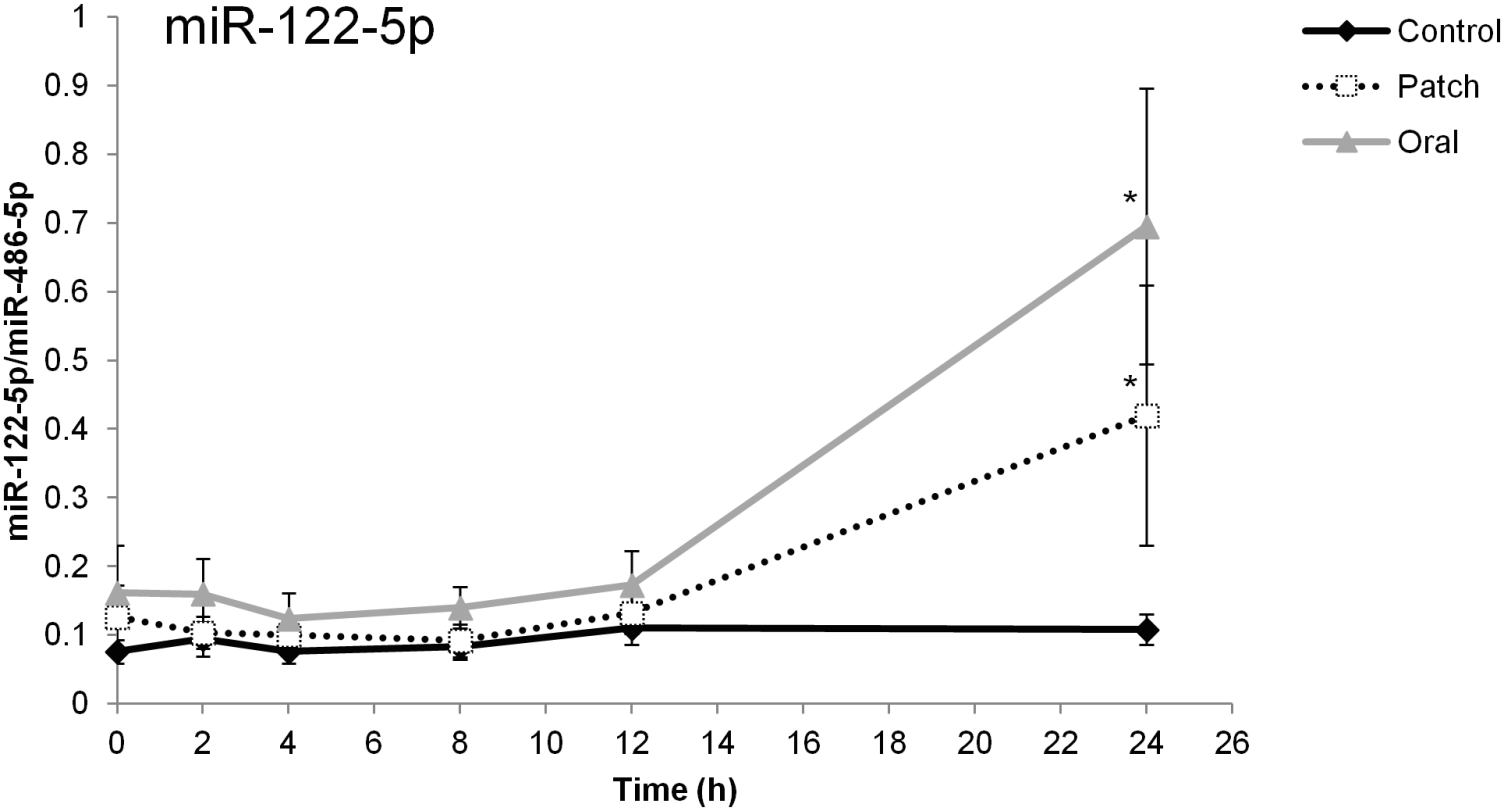
Circulating miR-122 levels after oral or transdermal T administration and during the control period. Level of plasma miR-122 after ingestion of two TU tablets (gray line) and application of two Testopatches (dashed line) or in the control period (black line). Data were normalized against the corresponding levels of endogenous miR-486-5p Collection time points are indicated on the x-axis. Values are expressed as means (±SE) of 19 independent samples. (*) indicates statistically significant difference relative to time = 0 h and the control.

T administration induced miR-122 with a peak occurring 24 h after drug intake (Fig 2). Consistent with the screening results, the increase in plasma miR-122 level was evident only at 24 h after T administration. Exogenous testosterone increased the levels of this circulating miRNA by an average of 4-fold. The amplitude of variation between baseline and 24 h was larger after ingestion of TU than after application of diffusion patches (Fig 2). During the control phase, the plasma level of miR-122 did not change significantly.

**Fig 3:**
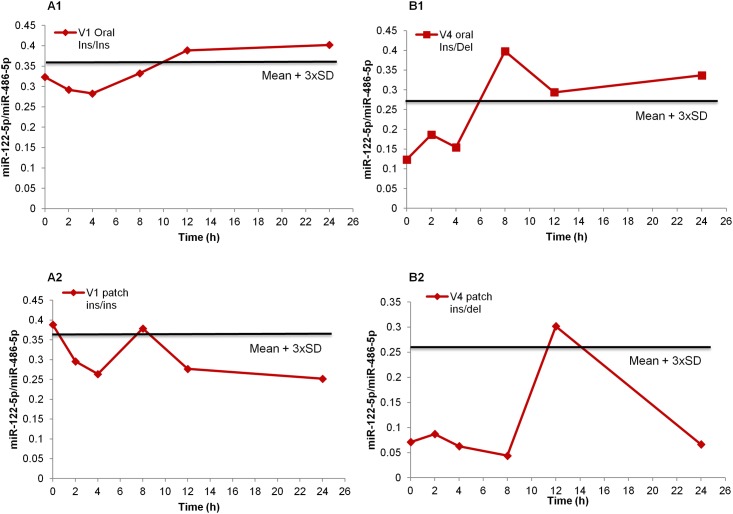
Examples of individual follow-up of miR-122 with personalized threshold. **(A and B)** Two examples of longitudinal monitoring of plasma miR-122 with personalized threshold (indicated by continuous black line) for two volunteers with different *UGT2B17* genotypes (ins/del and ins/ins) after oral and transdermal intake of T. Thresholds are calculated as the mean of the control phase results + 3 × SD. In these examples, longitudinal monitoring of plasma miR-122 levels after oral T administration is more sensitive than of transdermal T administration. Data were normalized against the corresponding levels of endogenous miR-486-5p.

Circulating miR-122 level was integrated in an individual longitudinal follow-up setting subject-based threshold (Fig 3). For each subject, the threshold was calculated as the mean + three standard deviations (SD) of the values during the control phase [6, 7, 30]. Fig 3 shows measurements of miR-122 levels over time in two volunteers with different *UGT2B17* genotypes following oral T administration, along with their individual thresholds.

Because miR-122 is highly expressed by the liver, the activity of hepatocellular enzymes (ALT/AST) was also determined in the serum of volunteers. In these analyses, the focus was placed on samples that exhibited a large increase of plasma miR-122 following T administration. However, longitudinal monitoring revealed no significant changes in either enzyme over time (S2 Fig). Likewise, no significant change was observed in CRP, a biochemical marker of leukocytes, after T administration (S2 Fig).

**Fig 4:**
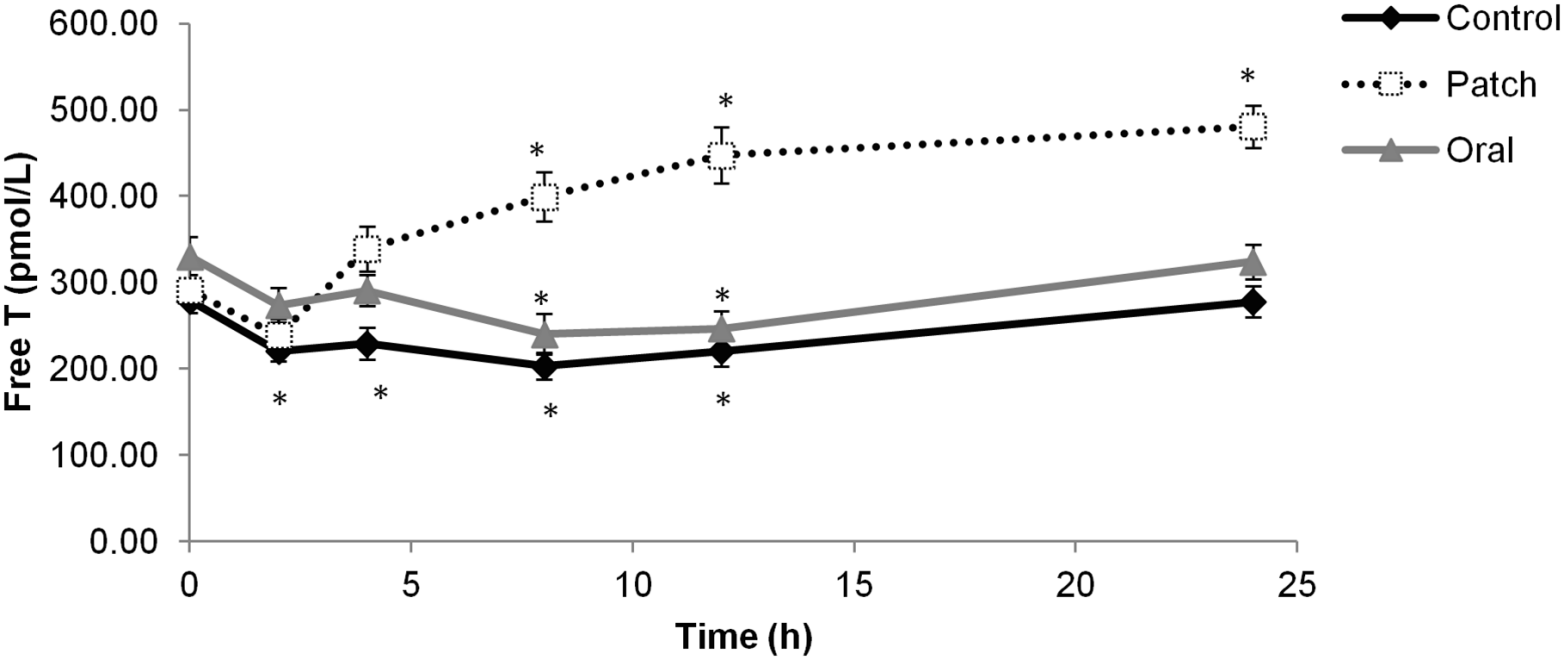
Serum free T concentrations after oral or transdermal T administration and during the control period. Mean (±SE) serum free T after oral (gray line) or transdermal (dashed line) T administration or in the control phase (black line). Values represent the average of 19 independent samples at each time point. (*) indicates statistically significant difference relative to time = 0 h.

Because intake of exogenous T is thought to affect endogenous T concentration, serum free T concentrations of volunteers were measured by immunoassay under each condition (Fig 4). A clear increase in serum free T was apparent from 8 to 24 h after transdermal T administration. However, ingestion of oral T did not affect serum free T, which followed the same pattern as during the control period (significant decrease at 8 and 12 h, returning to baseline value at 24 h).

## Discussion

In this study, we investigated whether circulating miRNAs could be used to detect testosterone abuse. Our key finding was that an exogenous source of testosterone triggered an increase in the level of a specific miRNA relative to a control period during which no treatment was administered. This observation suggests that this circulating miRNAs may serve as biomarkers to detect exogenous testosterone intake.

Our screen for potential circulating miRNAs whose levels were altered by testosterone identified three candidates that might serve as efficient biomarkers of testosterone administration via different dosage routes. These candidates were confirmed by individual qPCR assays, and their kinetics were compared with the control results.

Administration of T via either of two routes triggered a significant decrease in the levels of miR-342 and miR-150. These miRNAs are mainly expressed in white blood cells, and miR-150 is particularly abundant in mature B and T lymphocytes [31, 32]. Levels of both miRNAs decreased markedly relative to baseline (0 h) 1 day after T administration. WBC count did not change significantly over the course of the study, eliminating this parameter as an explanatory variable. One potential explanation for this decrease is the immunosuppressive effect of T, which might decrease the rate of release of miRNAs from WBCs [33–35]. However, the kinetics of natural variations in levels of miR-342 and miR-150 (control) were very similar to the kinetics of variation during the testosterone phases, especially between 12 and 24 h. The control and testosterone phases differed primarily in regard to the baseline levels of miR-342 and miR-150, which were higher during both intervention periods. Therefore, we cannot conclude that the decrease in both miRNAs was testosterone-dependent, so they cannot serve as biomarkers for the detection of testosterone. Further investigations should be conducted to characterize the natural variations in the plasma levels of these miRNAs over periods longer than 24 h.

Intake of testosterone, either orally or topically, increased the plasma level of only one miRNA, which was miR-122. Unlike miR-342 and miR-150, its expression level remained constant throughout the day during the control period, supporting the idea that the peak induction of plasma miR-122 1 day after T intake was specific to the performance-enhancing drug rather than the circadian rhythm. miR-122 is expressed primarily in the liver, where it regulates lipid metabolism [26, 36, 37], and it has already been investigated as a potential biomarker of drug-induced liver injury [26, 37].

The liver is a target organ of testosterone, which influences liver metabolism, suppresses hepatic protective responses, and promotes hepatocellular carcinoma [38–40]. However, very little information is available regarding the effect T on hepatic miRNA expression. Delic et al reported upregulation of six miRNAs, including miR-122, in female mouse liver following 3 weeks of T treatment [41]. *In silico* analysis identified an androgen response element (ARE) in the promoter region of miR-122, suggesting that T-induced upregulation of miR-122 is mediated by an interaction between the T-AR complex and its cognate response element. However, a more detailed understanding of the regulation and function of cell-free miRNAs is required before we can safely conclude that the increase in plasma miR-122 is the result of direct transcriptional activation by androgen nuclear receptor.

Alternatively, the increase of plasma miR-122 may be the result of T-induced hepatotoxicity, particularly when the drug is taken orally. However, CRP and AST/ALT levels did not vary significantly, eliminating hepatic inflammation as an explanatory factor. Instead, given that circulating miRNAs may be byproducts of hepatic cells, an increase in cellular activity might underlie the alterations in release of miRNAs by the liver [21, 42, 43]. Consistent with this idea, T administered by various routes is metabolized in the liver, increasing hepatocytes activity.

Even though a clear induction of circulating miR-122 was observed 1 day after oral or transdermal T administration, the sensitivity of this method was compromised by the high inter-individual variability, reflecting the heterogeneity of the volunteers in response to the intervention. Based on the idea of a transcriptomic passport, one appropriate approach would involve integration of miR-122 level into an individual longitudinal follow-up with a personalized threshold. To this end, we applied a method that previously yielded successful results in steroid profiling [6, 7, 30]. In this method, the mean ± 3 × SD of the control values for a given volunteer was established as his personalized threshold.

This method has several advantages over previous approaches. First, in contrast to classical urine biomarkers such as the T/E ratio, commonly used in accredited laboratories, it is independent of the *UGT2B17* genotype of the subject. Second, expression of plasma miR-122 is independent of blood-cell count [36]. Third, the detection window is also longer than that of individual monitoring of typical urinary metabolites, especially in the context of oral T administration, which is between 2 h and 12 h. However, because a single biomarker is not sufficiently powerful, circulating miR-122 should be combined with several other biomarkers to increase the sensitivity of testosterone screening.

Recently, a new approach based on individual follow-up of blood steroid hormones by LC-MS/MS was proposed as a complement to urinary steroid profiling [44]. This method, which is also independent of *UGT2B17* genotype, has the additional advantage that blood matrix is more stable and less susceptible to confounding factors than urine. However, although LC-MS/MS effectively detected T after transdermal application, longitudinal monitoring after oral TU misuse was less sensitive.

In agreement with this finding, no variation of serum free T was observed after oral TU ingestion in our study. This surprising observation could be explained by a rapid metabolism of free T into 5α-dihydrotestosterone, androstenedione and estradiol [45]. Indeed, two studies reported T retro-conversion to androstenedione following 50 mg oral DHEA or 20 mg androstenedione sublingual ciclodextrin tablet in young males [46, 47]. Finally, food has a particular importance on the bioavailability of oral TU [48].

On the other side, oral TU ingestion triggered a clear increase in plasma miR-122 with a larger amplitude than that observed after transdermal administration and a detection window longer than those of typical urinary biomarkers. The difference between both routes of administration could be explained by the first-pass effect after oral T administration, associated with a great increase of hepatocytes activity and miR-122 secretion. In contrast, transdermal systems slowly deliver T in the body leading to a weaker activity of the liver and a less important secretion of miR-122.

This finding opens the door to using the blood steroid profile in combination with circulating miRNA signatures to increase the sensitivity of the approach [44]. This integration of multiple parameters, transcriptomic and metabolomic, in a longitudinal follow-up can improve the discriminatory power of T detection, and thus represents a powerful and efficient tool for targeting athlete samples for GC-C-IRMS testing. Because both methods require blood samples, the collection process is simplified, and no additional sample material must to be taken from the athlete.

To address issues of reproducibility and robustness, we chose an endogenous control to counteract the effects of physiological variation in miRNA levels between individuals. To this end, we identified the least variable miRNAs by screening; miR-486-5p was detected as a circulating miRNAs whose abundance was not altered by testosterone via either route of administration. This miRNA is highly expressed in RBCs [36], is present at high levels in plasma (27.5 Ct), and was already shown to be an efficient endogenous control in different studies [21, 49]. Therefore, we chose miR-486-5p as an endogenous control for data normalization of relative expression.

Variations between studies are also related to choice of blood medium, potentially due to intercellular miRNA trafficking during the coagulation process [19]. In our study, results were inconsistent between plasma and serum. Expression levels were particularly low in serum, and our profiling screen was unable to identify any miRNAs that were differentially expressed after oral ingestion of TU (S4 Table).

The study had several non-negligible limitations. To integrate circulating miRNAs as biomarkers into the adaptive model of the ABP, intrinsic and extrinsic factors that might affect measurements of cell-free miRNAs must be fully characterized. In addition, the influence of confounding factors such as sex, age, high altitude, or physical exercise remains to be determined. Long-term longitudinal measurement of miR-122 in elite athletes and controls is also required before this circulating miRNA can be used in an anti-doping context. Monitoring of plasma miR-122 for a longer period than 24 h would also have been of particular interest.

In summary, our results suggest that transcriptomic biomarkers may be used for detection of T administered either orally or transdermally. This steroid induced an increase of a specific miRNA whose origin was related to the liver. Nevertheless, due to high inter-individual variability, longitudinal and individual measurement was the optimal strategy. When using the Athlete Biological Passport, a suspicion of doping is based on a combination of several biomarkers. Therefore, the addition of circulating miRNAs to the adaptive model has the potential to enhance its discriminatory performance. Our study thus provides the basis for using a combination of steroidomic and transcriptomic biomarkers as a new approach that is complementary to urine-based methods for detection of testosterone and steroids.

## Supporting Information

S1 Fig Study design. Samples were collected at different time points after application of two Testopatches (patch phase) at week 2 and the ingestion of two TU tablets (oral phase) at week 5 after a wash-out period of 2 weeks. During the first week of the study, samples were also gathered at the same time points with no treatment (control phase). Circulating miRNAs were extracted from plasma or serum, reverse transcribed into cDNA and quantitated by RT-qPCR. Time points selected for the screening are indicated in bold.
(TIF)

S2 Fig AST/ALT and CRP levels after oral or transdermal T administration or during the control period. **(A and B)**. Mean (±SE) ALT and AST activity (U/I) at the indicated time points after testosterone administration. Values represent the average of seven independent samples (for patch phase) or ten independent samples (for oral phase). **(C)** Mean (±SE) CRP (mg/dl) at the indicated time points during each phase. Values represent the average of 19 independent samples at each time point.
(TIF)

S1 Table Ct values of plasma miRNAs at 0 h and 24 h for each subject in both intervention periods. (XLSX)

S2 Table Total affected plasma miRNAs by oral and transdermal T administration. (XLSX)

S3 Table Least variable miRNAs. List of circulating miRNAs whose levels were invariant following oral or transdermal T administration.
(PDF)

S4 Table Ct values of serum miRNAs at 0 h and 24 h for each subject in oral period. (XLSX)

## References

[1] Eberhard Nieschlag HMB, Susan Nieschlag. Testosterone: Action, Deficiency, Substitution. 2012.

[2] Senmaru T, Fukui M, Okada H, Mineoka Y, Yamazaki M, Tsujikawa M, et al Testosterone deficiency induces markedly decreased serum triglycerides, increased small dense LDL, and hepatic steatosis mediated by dysregulation of lipid assembly and secretion in mice fed a high-fat diet. Metabolism: clinical and experimental. 2013;62(6):851–60. 10.1016/j.metabol.2012.12.007 . 23332447

[3] World Anti-Doping Agency (WADA). The prohibited list 2015, International standard. 2015. https://wada-main-prod.s3.amazonaws.com/resources/files/wada-2015-prohibited-list-en.pdf

[4] Saudan C, Baume N, Robinson N, Avois L, Mangin P, Saugy M. Testosterone and doping control. British journal of sports medicine. 2006;40 Suppl 1:i21–4. 10.1136/bjsm.2006.027482 16799097 PMC2657495

[5] World Anti-Doping Agency (WADA). Reporting and Evaluation Guidance for testosterone, epitestosterone, T/E ratio and other endogenous steroids: WADA; 2004 [cited June 2015].

[6] Badoud F, Boccard J, Schweizer C, Pralong F, Saugy M, Baume N. Profiling of steroid metabolites after transdermal and oral administration of testosterone by ultra-high pressure liquid chromatography coupled to quadrupole time-of-flight mass spectrometry. The Journal of steroid biochemistry and molecular biology. 2013;138:222–35. 10.1016/j.jsbmb.2013.05.018 . 23796409

[7] Fabregat A, Pozo OJ, Marcos J, Segura J, Ventura R. Alternative markers for the long-term detection of oral testosterone misuse. Steroids. 2011;76(12):1367–76. 10.1016/j.steroids.2011.07.005 . 21782838

[8] Strahm E, Sottas PE, Schweizer C, Saugy M, Dvorak J, Saudan C. Steroid profiles of professional soccer players: an international comparative study. British journal of sports medicine. 2009;43(14):1126–30. 10.1136/bjsm.2008.056242 . 19282302

[9] World Anti-Doping Agency (WADA). ABP Operating Guidelines. 2014. https://wada-main-prod.s3.amazonaws.com/resources/files/wada-abp-operating-guidelines-v5.0-en.pdf

[10] Kuuranne T, Saugy M, Baume N. Confounding factors and genetic polymorphism in the evaluation of individual steroid profiling. British journal of sports medicine. 2014;48(10):848–55. 10.1136/bjsports-2014-093510 24764553 PMC4033181

[11] Juul A, Sorensen K, Aksglaede L, Garn I, Rajpert-De Meyts E, Hullstein I, et al A common deletion in the uridine diphosphate glucuronyltransferase (UGT) 2B17 gene is a strong determinant of androgen excretion in healthy pubertal boys. The Journal of clinical endocrinology and metabolism. 2009;94(3):1005–11. 10.1210/jc.2008-1984 . 19088161

[12] Rane A, Ekstrom L. Androgens and doping tests: genetic variation and pit-falls. British journal of clinical pharmacology. 2012;74(1):3–15. 10.1111/j.1365-2125.2012.04294.x 22506612 PMC3394124

[13] Kavitha N, Vijayarathna S, Jothy SL, Oon CE, Chen Y, Kanwar JR, et al MicroRNAs: biogenesis, roles for carcinogenesis and as potential biomarkers for cancer diagnosis and prognosis. Asian Pacific journal of cancer prevention: APJCP. 2014;15(18):7489–97. . 25292018 10.7314/apjcp.2014.15.18.7489

[14] Hata A, Lieberman J. Dysregulation of microRNA biogenesis and gene silencing in cancer. Science signaling. 2015;8(368):re3 10.1126/scisignal.2005825 . 25783160

[15] Falcone G, Felsani A, D'Agnano I. Signaling by exosomal microRNAs in cancer. Journal of experimental & clinical cancer research: CR. 2015;34(1):32 10.1186/s13046-015-0148-3 25886763 PMC4391656

[16] Weber JA, Baxter DH, Zhang S, Huang DY, Huang KH, Lee MJ, et al The microRNA spectrum in 12 body fluids. Clinical chemistry. 2010;56(11):1733–41. 10.1373/clinchem.2010.147405 . 20847327 PMC4846276

[17] Reid G, Kirschner MB, van Zandwijk N. Circulating microRNAs: Association with disease and potential use as biomarkers. Critical reviews in oncology/hematology. 2011;80(2):193–208. 10.1016/j.critrevonc.2010.11.004 . 21145252

[18] Shi Q, Yang X. Circulating MicroRNA and Long Noncoding RNA as Biomarkers of Cardiovascular Diseases. Journal of cellular physiology. 2015 10.1002/jcp.25174 . 26308238

[19] He Y, Lin J, Kong D, Huang M, Xu C, Kim TK, et al Current State of Circulating MicroRNAs as Cancer Biomarkers. Clinical chemistry. 2015;61(9):1138–55. 10.1373/clinchem.2015.241190 . 26319452

[20] Leuenberger N, Jan N, Pradervand S, Robinson N, Saugy M. Circulating microRNAs as long-term biomarkers for the detection of erythropoiesis-stimulating agent abuse. Drug testing and analysis. 2011;3(11–12):771–6. 10.1002/dta.370 . 22113880

[21] Leuenberger N, Schumacher YO, Pradervand S, Sander T, Saugy M, Pottgiesser T. Circulating microRNAs as biomarkers for detection of autologous blood transfusion. PloS one. 2013;8(6):e66309 10.1371/journal.pone.0066309 23840438 PMC3688786

[22] Kelly BN, Haverstick DM, Lee JK, Thorner MO, Vance ML, Xin W, et al Circulating microRNA as a biomarker of human growth hormone administration to patients. Drug testing and analysis. 2014;6(3):234–8. 10.1002/dta.1469 . 23495241

[23] Leuenberger N, Robinson N, Saugy M. Circulating miRNAs: a new generation of anti-doping biomarkers. Analytical and bioanalytical chemistry. 2013;405(30):9617–23. 10.1007/s00216-013-7340-0 . 24077830

[24] Gilad S, Meiri E, Yogev Y, Benjamin S, Lebanony D, Yerushalmi N, et al Serum microRNAs are promising novel biomarkers. PloS one. 2008;3(9):e3148 10.1371/journal.pone.0003148 18773077 PMC2519789

[25] Mitchell PS, Parkin RK, Kroh EM, Fritz BR, Wyman SK, Pogosova-Agadjanyan EL, et al Circulating microRNAs as stable blood-based markers for cancer detection. Proceedings of the National Academy of Sciences of the United States of America. 2008;105(30):10513–8. 10.1073/pnas.0804549105 18663219 PMC2492472

[26] Wang K, Zhang S, Marzolf B, Troisch P, Brightman A, Hu Z, et al Circulating microRNAs, potential biomarkers for drug-induced liver injury. Proceedings of the National Academy of Sciences of the United States of America. 2009;106(11):4402–7. 10.1073/pnas.0813371106 19246379 PMC2657429

[27] Andreasen D, Fog JU, Biggs W, Salomon J, Dahslveen IK, Baker A, et al Improved microRNA quantification in total RNA from clinical samples. Methods. 2010;50(4):S6–9. 10.1016/j.ymeth.2010.01.006 . 20215018

[28] Cheng HH, Yi HS, Kim Y, Kroh EM, Chien JW, Eaton KD, et al Plasma processing conditions substantially influence circulating microRNA biomarker levels. PloS one. 2013;8(6):e64795 10.1371/journal.pone.0064795 23762257 PMC3676411

[29] McDonald JS, Milosevic D, Reddi HV, Grebe SK, Algeciras-Schimnich A. Analysis of circulating microRNA: preanalytical and analytical challenges. Clinical chemistry. 2011;57(6):833–40. 10.1373/clinchem.2010.157198 . 21487102

[30] Fabregat A, Pozo OJ, Van Renterghem P, Van Eenoo P, Marcos J, Segura J, et al Detection of dihydrotestosterone gel, oral dehydroepiandrosterone, and testosterone gel misuse through the quantification of testosterone metabolites released after alkaline treatment. Drug testing and analysis. 2011;3(11–12):828–35. 10.1002/dta.351 . 21998068

[31] Merkerova M, Belickova M, Bruchova H. Differential expression of microRNAs in hematopoietic cell lineages. European journal of haematology. 2008;81(4):304–10. 10.1111/j.1600-0609.2008.01111.x . 18573170

[32] He Y, Jiang X, Chen J. The role of miR-150 in normal and malignant hematopoiesis. Oncogene. 2014;33(30):3887–93. 10.1038/onc.2013.346 . 23955084

[33] McMurray RW, Suwannaroj S, Ndebele K, Jenkins JK. Differential effects of sex steroids on T and B cells: modulation of cell cycle phase distribution, apoptosis and bcl-2 protein levels. Pathobiology: journal of immunopathology, molecular and cellular biology. 2001;69(1):44–58. 48757. . 11641617 10.1159/000048757

[34] Gutsol AA, Sokhonevich NA, Kofanova KA, Litvinova LS. [The influence of testosterone and beta-estradiol on T-lymphocytes activation associated with IL-2 production and expression of CD25 (IL-2Ralpha) molecules]. Tsitologiia. 2014;56(7):500–3. . 25696993

[35] Trigunaite A, Dimo J, Jorgensen TN. Suppressive effects of androgens on the immune system. Cellular immunology. 2015;294(2):87–94. 10.1016/j.cellimm.2015.02.004 . 25708485

[36] Pritchard CC, Kroh E, Wood B, Arroyo JD, Dougherty KJ, Miyaji MM, et al Blood cell origin of circulating microRNAs: a cautionary note for cancer biomarker studies. Cancer prevention research. 2012;5(3):492–7. 10.1158/1940-6207.CAPR-11-0370 22158052 PMC4186243

[37] Starkey Lewis PJ, Dear J, Platt V, Simpson KJ, Craig DG, Antoine DJ, et al Circulating microRNAs as potential markers of human drug-induced liver injury. Hepatology. 2011;54(5):1767–76. 10.1002/hep.24538 . 22045675

[38] Krücken J DM, Braun JV, Schroetel RM, El-Khadragy M, Carmeliet P, Mossmann H, Wunderlich F. Testosterone suppresses protective responses of the liver to blood-stage malaria. Infection and Immunity. 2005;73:436–43. PubMed Central PMCID: PMCPMC538982 15618182 10.1128/IAI.73.1.436-443.2005 PMC538982

[39] Solbach P, Potthoff A, Raatschen HJ, Soudah B, Lehmann U, Schneider A, et al Testosterone-receptor positive hepatocellular carcinoma in a 29-year old bodybuilder with a history of anabolic androgenic steroid abuse: a case report. BMC gastroenterology. 2015;15:60 10.1186/s12876-015-0288-0 25986067 PMC4461943

[40] Yu MW, Chen CJ. Elevated serum testosterone levels and risk of hepatocellular carcinoma. Cancer research. 1993;53(4):790–4. . 8381328

[41] Delic D, Grosser C, Dkhil M, Al-Quraishy S, Wunderlich F. Testosterone-induced upregulation of miRNAs in the female mouse liver. Steroids. 2010;75(12):998–1004. 10.1016/j.steroids.2010.06.010 . 20600201

[42] Turchinovich A, Weiz L, Burwinkel B. Extracellular miRNAs: the mystery of their origin and function. Trends in biochemical sciences. 2012;37(11):460–5. 10.1016/j.tibs.2012.08.003 . 22944280

[43] Turchinovich A, Weiz L, Langheinz A, Burwinkel B. Characterization of extracellular circulating microRNA. Nucleic acids research. 2011;39(16):7223–33. 10.1093/nar/gkr254 21609964 PMC3167594

[44] Ponzetto F, Mehl F, Boccard J, Baume N, Rudaz S, Saugy M, et al Longitudinal monitoring of endogenous steroids in human serum by UHPLC-MS/MS as a tool to detect testosterone abuse in sports. Analytical and bioanalytical chemistry. 2015 10.1007/s00216-015-9185-1 . 26677027

[45] Merck Canada Inc. http://www.merck.ca/assets/en/pdf/products/ANDRIOL-PM_E.pdf. 2016 [cited 2016].

[46] Brown GA, Vukovich MD, Sharp RL, Reifenrath TA, Parsons KA, King DS. Effect of oral DHEA on serum testosterone and adaptations to resistance training in young men. J Appl Physiol (1985). 1999;87(6):2274–83. . 10601178 10.1152/jappl.1999.87.6.2274

[47] Brown GA, Martini ER, Roberts BS, Vukovich MD, King DS. Acute hormonal response to sublingual androstenediol intake in young men. J Appl Physiol (1985). 2002;92(1):142–6. . 11744653 10.1152/jappl.2002.92.1.142

[48] Bagchus WM, Hust R, Maris F, Schnabel PG, Houwing NS. Important effect of food on the bioavailability of oral testosterone undecanoate. Pharmacotherapy. 2003;23(3):319–25. . 12627930 10.1592/phco.23.3.319.32104

[49] Boeri M, Verri C, Conte D, Roz L, Modena P, Facchinetti F, et al MicroRNA signatures in tissues and plasma predict development and prognosis of computed tomography detected lung cancer. Proceedings of the National Academy of Sciences of the United States of America. 2011;108(9):3713–8. 10.1073/pnas.1100048108 21300873 PMC3048155

