# Supplementary figures and images for: Circulating microRNA-122 as Potential Biomarker for Detection of Testosterone Abuse
Source: PLoS One. 2016 May 12;11(5):e0155248. doi: 10.1371/journal.pone.0155248 (PMC4865044; doi:10.1371/journal.pone.0155248)

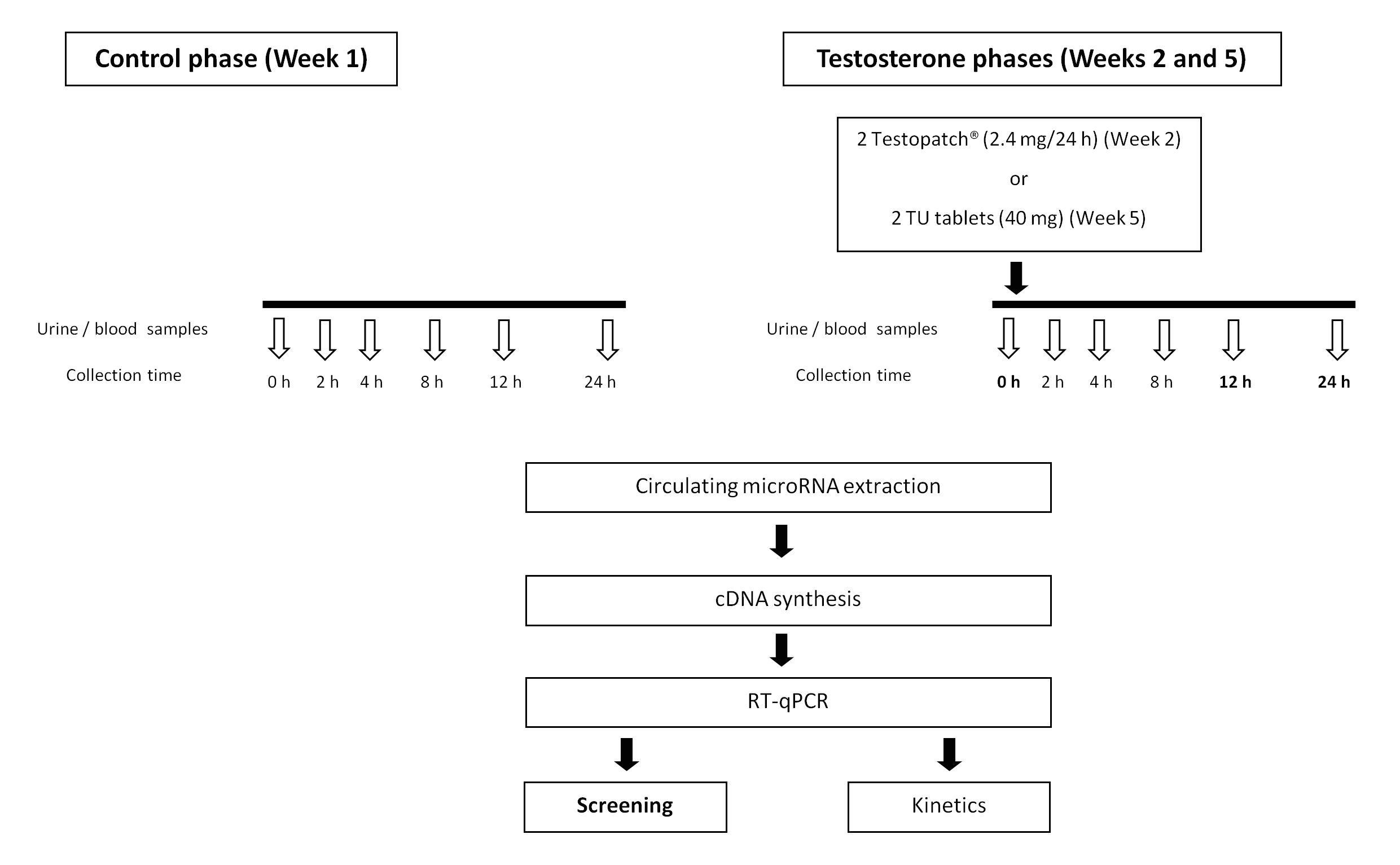

Supplement: S1 Fig — Samples were collected at different time points after application of two Testopatches (patch phase) at week 2 and the ingestion of two TU tablets (oral phase) at week 5 after a wash-out period of 2 weeks. During the first week of the study, samples were also gathered at the same time points with no treatment (control phase). Circulating miRNAs were extracted from plasma or serum, reverse transcribed into cDNA and quantitated by RT-qPCR. Time points selected for the screening are indicated in bold. (TIF) [file pone.0155248.s001.TIF]

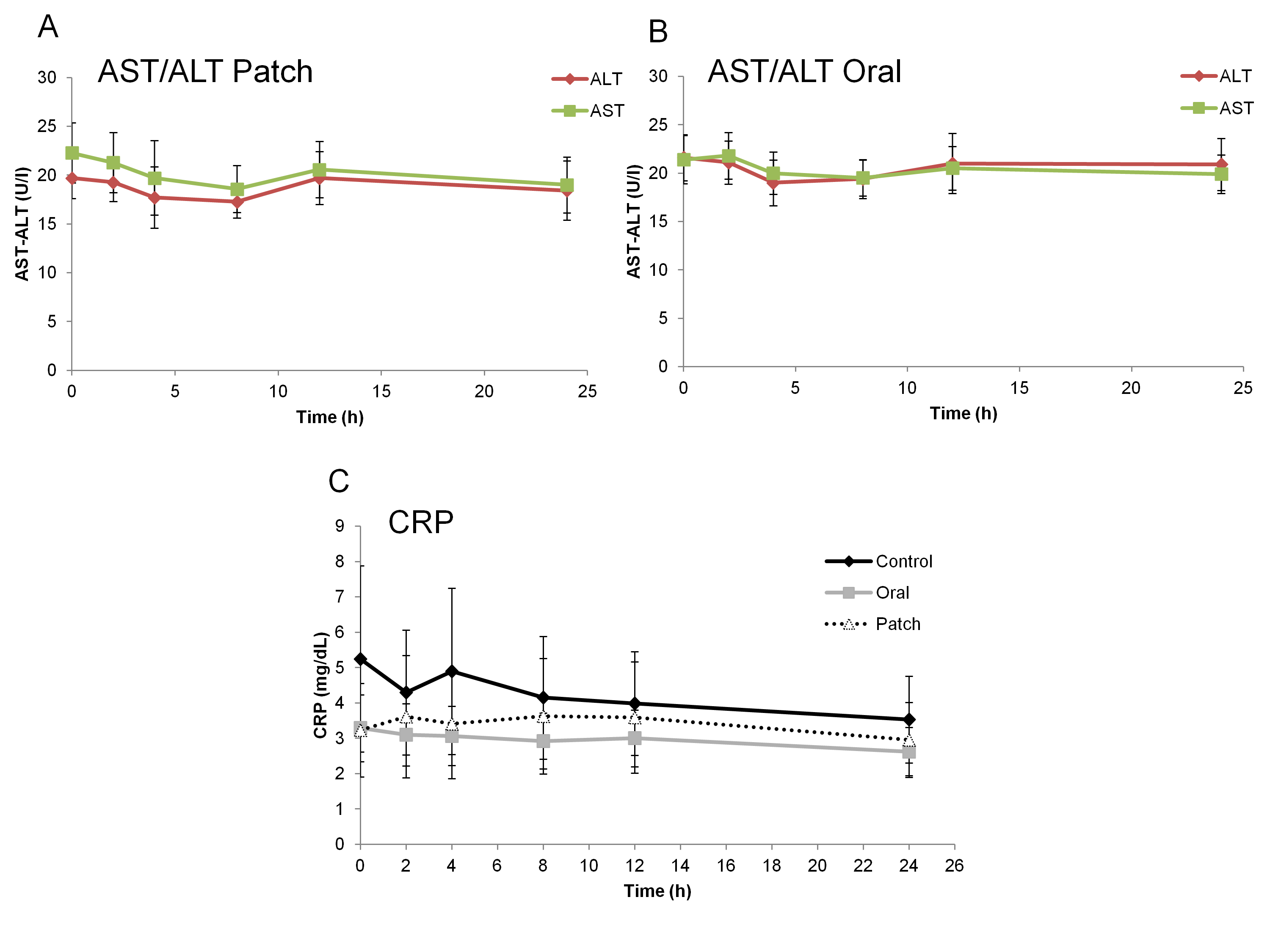

Supplement: S2 Fig — (A and B). Mean (±SE) ALT and AST activity (U/I) at the indicated time points after testosterone administration. Values represent the average of seven independent samples (for patch phase) or ten independent samples (for oral phase). (C) Mean (±SE) CRP (mg/dl) at the indicated time points during each phase. Values represent the average of 19 independent samples at each time point. (TIF) [file pone.0155248.s002.tif]
