# Supplementary material for: Circulating microRNA-122 as Potential Biomarker for Detection of Testosterone Abuse
Source: PLoS One. 2016 May 12;11(5):e0155248. doi: 10.1371/journal.pone.0155248 (PMC4865044; doi:10.1371/journal.pone.0155248)
Supplement: S3 Table — List of circulating miRNAs whose levels were invariant following oral or transdermal T administration. (PDF) [file pone.0155248.s005.pdf]

S3 Table. Least variable miRNAs. List of circulating miRNAs whose levels were invariant following oral or transdermal T administration

Oral Testosterone			
**miRNA**	**Mean Ct**	**SD Ct**	
UniSp3-3	20.69	0.153225	Control
UniSp3-2	20.67	0.196948	Control
UniSp6	20.34	0.427376	Control
UniSp3-1	20.72	0.536442	Control
hsa-miR-92a-3p	26.39	0.721265	
hsa-miR-320a	28.39	0.768094	
hsa-miR-185-5p	27.15	0.793265	
hsa-miR-15a-5p	26.9	0.810257	
hsa-miR-106a-5p	27.67	0.824582	
hsa-miR-16-5p	22.48	0.845336	
**hsa-miR-486-5p**	**27.35**	**0.855301**	
hsa-miR-126-3p	28.76	0.926652	
hsa-miR-93-5p	28.86	0.953129	
hsa-miR-25-3p	27.95	0.968228	
hsa-miR-21-5p	29.23	0.973654	
hsa-miR-451a	23.56	0.978079	
hsa-miR-19b-3p	26.93	0.9975	

Transdermal Testosterone			
**miRNA**	**Mean Ct**	**SD Ct**	
UniSp3-3	20	0.075313	Control
UniSp3-1	20.01	0.104053	Control
UniSp3-2	19.86	0.11639	Control
UniSp6	19.54	0.189347	Control
cel-miR-39-3p	29.97	0.381224	Control
UniSp2	21.88	0.387459	Control
UniSp4	28.92	0.699474	Control
hsa-miR-185-5p	27.58	0.73912	
hsa-miR-126-3p	28.79	0.750153	
hsa-miR-92a-3p	26.96	0.784239	
hsa-miR-20a-5p	27.12	0.80739	
**hsa-miR-486-5p**	**27.78**	**0.815407**	
hsa-miR-16-5p	22.61	0.830682	
hsa-miR-106a-5p	28.09	0.836233	
hsa-miR-320a	29.04	0.839432	
hsa-miR-21-5p	27.18	0.857388	
hsa-miR-19a-3p	27.6	0.865613	
hsa-miR-25-3p	27.95	0.871034	
hsa-miR-140-3p	29	0.916779	
hsa-miR-19b-3p	26.26	0.934286	
hsa-miR-93-5p	28.32	0.984927	
hsa-miR-103a-3p	29.85	0.993937	

* The miRNA that was chosen as an endogenous control for data normalization is indicated in bold and synthetic spikes-in are named “control”.
